# Bacteriophage Genetic Edition Using LSTM

**DOI:** 10.3389/fbinf.2022.932319

**Published:** 2022-07-13

**Authors:** Shabnam Ataee, Xavier Brochet, Carlos Andrés Peña-Reyes

**Affiliations:** ^1^ Institute of Information and Communication Technology (IICT), School of Management and Engineering Vaud (HEIG-VD), Yverdon-les-Bains, Switzerland; ^2^ HES-SO University of Applied Sciences and Arts Western Switzerland, Delémont, Switzerland; ^3^ CI4CB—Computational Intelligence for Computational Biology, SIB—Swiss Institute of Bioinformatics, Lausanne, Switzerland

**Keywords:** phage therapy, genome sequences, deep learning, 1D-CNN, LSTM, generative model, genetically engineered phages

## Abstract

Bacteriophages are gaining increasing interest as antimicrobial tools, largely due to the emergence of multi-antibiotic–resistant bacteria. Although their huge diversity and virulence make them particularly attractive for targeting a wide range of bacterial pathogens, it is difficult to select suitable phages due to their high specificity which limits their host range. In addition, other challenges remain such as structural fragility under certain environmental conditions, immunogenicity of phage therapy, or development of bacterial resistance. The use of genetically engineered phages may reduce characteristics that hinder prophylactic and therapeutic applications of phages. Nowadays, there is no systematic method to modify a given phage genome conferring its sought characteristics. We explore the use of artificial intelligence for this purpose as it has the potential to both guide and accelerate genome modification to generate phage variants with unique properties that overcome the limitations of natural phages. We propose an original architecture composed of two deep learning–driven components: a phage–bacterium interaction predictor and a phage genome-sequence generator. The former is a multi-branch 1-D convolutional neural network (1D-CNN) that analyses phage and bacterial genomes to predict interactions. The latter is a recurrent neural network, more particularly a long short-term memory (LSTM), that performs genomic modifications to a phage to offer substantial host range improvement. For this component, we developed two different architectures composed of one or two stacked LSTM layers with 256 neurons each. These generators are used to modify, more precisely to rewrite, the genome sequence of 42 selected phages, while the predictor is used to estimate the host range of the modified bacteriophages across 46 strains of *Pseudomonas aeruginosa*. The proposed generators, trained with an average accuracy of 96.1%, are able to improve the host range for an average of 18 phages among the 42 under study, increasing both their average host range, by 73.0 and 103.7%, and the maximum host ranges from 21 to 24 and 29, respectively. These promising results showed that the use of deep learning methodologies allows genetic modification of phages to extend, for instance, their host range, confirming the potential of these approaches to guide bacteriophage engineering.

## 1 Introduction

Phage therapy (PT) is a therapeutic approach to treat patients with bacterial infections. It is based on the use of viruses, called bacteriophages (or phages), to infect and kill pathogenic bacteria throughout their lifecycle (Matsuzaki et al., 2005). Present in all ecosystems, bacteriophages are viruses that naturally and specifically infect bacteria and are, therefore, unable to infect eukaryotic cells. This therapy was developed more than a century ago with the discovery of phages by researchers Frederick Twort (Twort, 1915) and Félix d’Herelle (1917) (D'Herelle, 2011). After promising successes, it was abandoned in favor of antibiotic therapy. However, in recent years, there has been a renewed interest in PT due to the emergence of nosocomial infections with antimicrobial-resistant (AMR) bacteria and the lack of new effective antibiotics. AMR is now considered by the World Health Organization as one of the greatest threats to global health, food security, and development [antibiotic resistance (WHO, 2021)]. This problem has been concretely measured both by the Centers for Disease Control (CDC), which reported more than 2.8 million antibiotic-resistant infections each year in the United States, causing more than 35,000 deaths (CDC, 2019) and by the United Kingdom, which commissioned a report on AMR in 2016 projecting to cause 10 million deaths per year by 2050. The concept of PT is to correctly match a bacterium with one or more phages capable of infecting and killing it.

After adsorption of the phage onto the target bacterium, the phage transfers its genome (viral nucleic acid, either DNA or RNA) into the bacterial cytoplasm. During the lytic cycle, the DNA is then transcribed, translated, and copied, to be assembled into viral particles (hijacking the bacterial replication machinery). In general, when a critical mass of viral particles is reached, bacterial lysis is actively triggered via lytic proteins that disrupt the bacterial wall, allowing the release of new viruses and leading to the death of the bacterium (Delbrück, 1940; Weinbauer, 2004). The advantages of PT are numerous and linked to the very nature of phages. These viruses, used as therapeutic agents, are able to regulate themselves, at sites of infection and once the bacteria are killed, phages do not replicate and can be rapidly eliminated by the immune system or other mechanisms.

It is estimated that there are about 10^31^ phages on Earth (Weitz et al., 2013; Mann, 2005) and they are in constant co-evolution with bacteria, which makes them a potentially inexhaustible source in nature. It is therefore theoretically possible to isolate new phages for most types of bacteria. This is important because new variants of pathogenic bacteria are appearing, leading to complicated therapeutic situations, especially with the emergence of multi-antibiotic–resistant bacteria. Moreover, the mechanisms of action of phages seem to be independent of those of antibiotics, and they do not provide selective pressure likely to increase antibiotic resistance. Finally, their narrow specificity against bacterial strains allows them to have a negligible impact on the patient’s microbiota (commensal flora). The host range of a phage is the spectrum or number of strains of bacterial species that a given specific phage can infect. Phages exhibit a narrow host range, and each phage can only infect a small number of bacteria, and therefore the use of a single phage has a low probability of being able to treat infections caused by several bacteria (Matsuzaki et al., 2014; Nilsson, 2014; Mapes et al., 2016). It is therefore essential to precisely identify the bacteria responsible for the infection before implementing the PT and to use a combination of several phages (phage cocktail) (Matsuzaki et al., 2005), which increases the number of targeted bacterial strains, that is, the spectrum of action of the treatment and reduces the rate of evolution of resistance to phages (Filippov et al., 2011; Gu et al., 2019; Ramirez et al., 2020).

However, the selection of adequate phages (discovery, isolation, and characterization) is time-consuming and requires laborious regulatory approval (Socher et al., 2011), which makes it one of the main limiting steps of PT. One forward-thinking modernization of phage therapy involves genetically modifying phages to overcome the limited efficiency of natural phages. In the last few years, the main genetic modifications applied to phages generally include i) mutations in genes (Yehl et al., 2019), ii) the partial or full replacement of genes (Mahichi et al., 2009; Lin et al., 2012; Dunne et al., 2019), and iii) the insertion of foreign genes (Bikard et al., 2014; Pei and Lamas-Samanamud, 2014; Yosef et al., 2015; Lam et al., 2021) using molecular techniques such as homologous recombination (HR) and genome rebooting with the aim of mainly improving the host range or enhancing the antibacterial effect of phages. For more details on the methodologies used by genetic engineering, we suggest recent reviews (Chen et al., 2019; Guo et al., 2021; Lenneman et al., 2021). All this research led to the first success in phage genetic engineering in 2019. In the context of the treatment of a 15-year-old lung transplant recipient (Dedrick et al., 2019), some genes were removed from phages to increase their activity. Such genetically engineered (GE) phages can therefore provide substantial advantages over natural phages in terms of host range, immune system recognition, and environmental stability. Phage engineering could provide a rapid strategy to generate phages with unique properties, and thus accelerate the development of PT, provided that a sound methodology is developed to suggest appropriate modifications to be applied to phage genomes. Currently, there is no such systematic method to guide the design of genetically engineered phages. Phages exhibit unparalleled genetic diversity, which makes it extremely complicated to factor in all possible variables for creating GE phages exhibiting expected properties. New technologies are needed to accelerate the design–build–test cycle for engineering phages and to make it possible to translate proof-of-concept academic work more efficiently into real-world use.

In this context, we explore herein the application of artificial intelligence (AI) which has the potential to both guide and accelerate genome modifications to generate phage variants with unique properties that overcome the limitations of natural phages. Until now, the application of AI to phage biology mainly concerns automated recovery, prediction, and classification of bacteriophages (Ren et al., 2017; Amgarten et al., 2018; Chibani et al., 2019; Kieft et al., 2020; Shang et al., 2021) predicting phage–bacteria interactions or host prediction at the genus, species, and/or strain levels (Leite et al., 2018; Ataee et al., 2020; Boeckaerts et al., 2021; Li et al., 2021; Zhou et al., 2022), bacteriophage lifecycle (McNair et al., 2012; Tynecki et al., 2020), or the identification of viral sequences (Seguritan et al., 2012; Manavalan et al., 2018; Cantu et al., 2020; Meng et al., 2020).

We propose a novel approach, dubbed PERPHECT (for Deep Generative Networks for Bacteriophage Genetic Edition), aiming to genetically engineer bacteriophages to enhance the activity of resulting phages (Ataee et al., 2020) (Ataee et al., 2020). The case study will focus on increasing the host range of phages. To achieve this, we combine state-of-the-art techniques from deep learning: a phagi–bacterium interaction predictor, the PERPHECT predictor, and a phage genome-sequence generator, the PERPHECT generator. Therefore, to evaluate the ability of our generator, we compare the host range of phages determined experimentally with their predicted host range computed by counting the number of bacterial strains predicted as positive by the PERPHECT predictor after rewriting their genomes. A key point of the PERPHECT architecture is that its two fundamental components are loosely coupled. With this property, different methods and approaches could be used to implement either or both the predictor and the generator.

In this article, we explore and evaluate the adequacy of a deep learning model to implement the generator component. For this purpose, we take advantage of LSTMs, a special type of recurrent neural network (Servan-Schreiber et al., 1988; Servan-Schreiber et al., 1991), to process long genomic sequences to capture high-level structures contained within them. LSTMs (Hochreiter and Schmidhuber, 1997; Sherstinsky, 2020) are extremely powerful deep learning models used to capture long-range dependencies since they are made of memory units allowing to save important features. LSTMs are particularly used in natural language processing (NLP), in applications such as paraphrase detection (Socher et al., 2011), speech recognition (Li et al., 2015), language modeling (Siami-Namini et al., 2018), text generation (Santhanam, 2018), but also for genome modeling (Li, 2019), or temporal data analysis (Abdel-Nasser and Mahmoud, 2017; Deng et al., 2020). The PERPHECT architecture paves the way for the use of deep learning methodologies to genetically modify phages and extend, for example, their host range, thus confirming the potential of these approaches to guide bacteriophage engineering.

## 2 Methods

**FIGURE 1:**
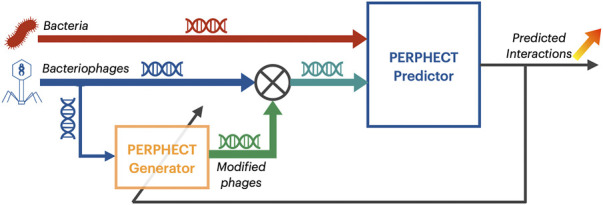
PERPHECT model architecture. PERPHECT architecture is formed of two fundamental components: i) The PERPHECT predictor used to predict potential interactions between bacteria and phages based solely on their genomic information and composed of a multi-branch 1-D CNN and ii) the PERPHECT generator used to modify existing phage genome sequences to improve their predicted host range and composed of LSTMs.

**FIGURE 2:**
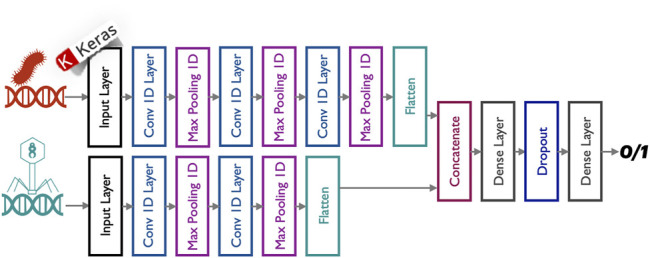
Phage–bacterium interaction predictor (Ataee, Rodriguez, Brochet, & Pena, 2020). The predictor model is composed of a stack of 1-D CNNs. The predictor architecture has a non-linear network topology. The two inputs (bacteria genome sequences and phages genome sequences) are processed separately by two parallel convolutional branches whose outputs are then merged and passed through two sub-sequential dense layers. A dropout layer is also used to reduce overfitting and to improve the generalization of the proposed deep neural network.

As already mentioned, the PERPHECT architecture is formed of two fundamental components (schematically represented in Figure 1):

• The PERPHECT predictor, used to predict potential interactions between bacteria and phages based solely on their genomic information, is a deep learning model, composed of a multi-branch 1-D CNN (Brownlee, 2018) (Figure 2). The proposed predictor was trained, validated, and tested on a dataset composed of 7,720 interactions between 227 bacteria and 3,208 phages. Its evaluation results showed performance figures as follows: 85% accuracy, 85% recall, 72% precision, and 78% f1-score on the test set. This predictor is explained in more detail (Ataee et al., 2020).
• The PERPHECT generator modifies existing phage genome sequences to improve their predicted host range. In this article, we concentrate on this component, presenting a novel model based on LSTM (Brownlee, 2017a), used to learn the context from the input sequence in order to make predictions. The model is conceived so that it is able to complete a genome sequence starting from a seed of *n* nucleotides and then predicting, iteratively one-by-one, the next nucleotides in the sequence.

### 2.1 Data

Among hundreds of bacterial genomes available in our dataset, we selected 46, belonging to the *Pseudomonas aeruginosa* species, as a target for phage infection. It is the species of the *Pseudomonas* genus that most often causes infections in humans. Unfortunately, many *Pseudomonas* infections are becoming difficult to treat as they are increasingly showing antibiotic resistance. Among all the interactions in our dataset, there are 42 phages able to infect at least one of the 46 *Pseudomonas aeruginosa* strains. We selected these phages as the, potentially, best candidates for genome modification. The genome sequences of 46 bacteria and 42 bacteriophages under study as well as the actual interaction between any bacterium and any phage under study are extracted from experimental results obtained by one of our partners during a previous project (Leite et al., 2018). Consequently, the new, specific dataset is composed of genome sequences for all 46 bacterial strains and 42 phages under study, as well as all the 1,932 interaction values, among which there are 277 positive interactions. In this dataset, our PERPHECT predictor exhibits a good performance when predicting interactions between the selected bacterial strains and their phages (accuracy=89%, recall=59%, specificity=95%, precision=68%) .

**FIGURE 3:**
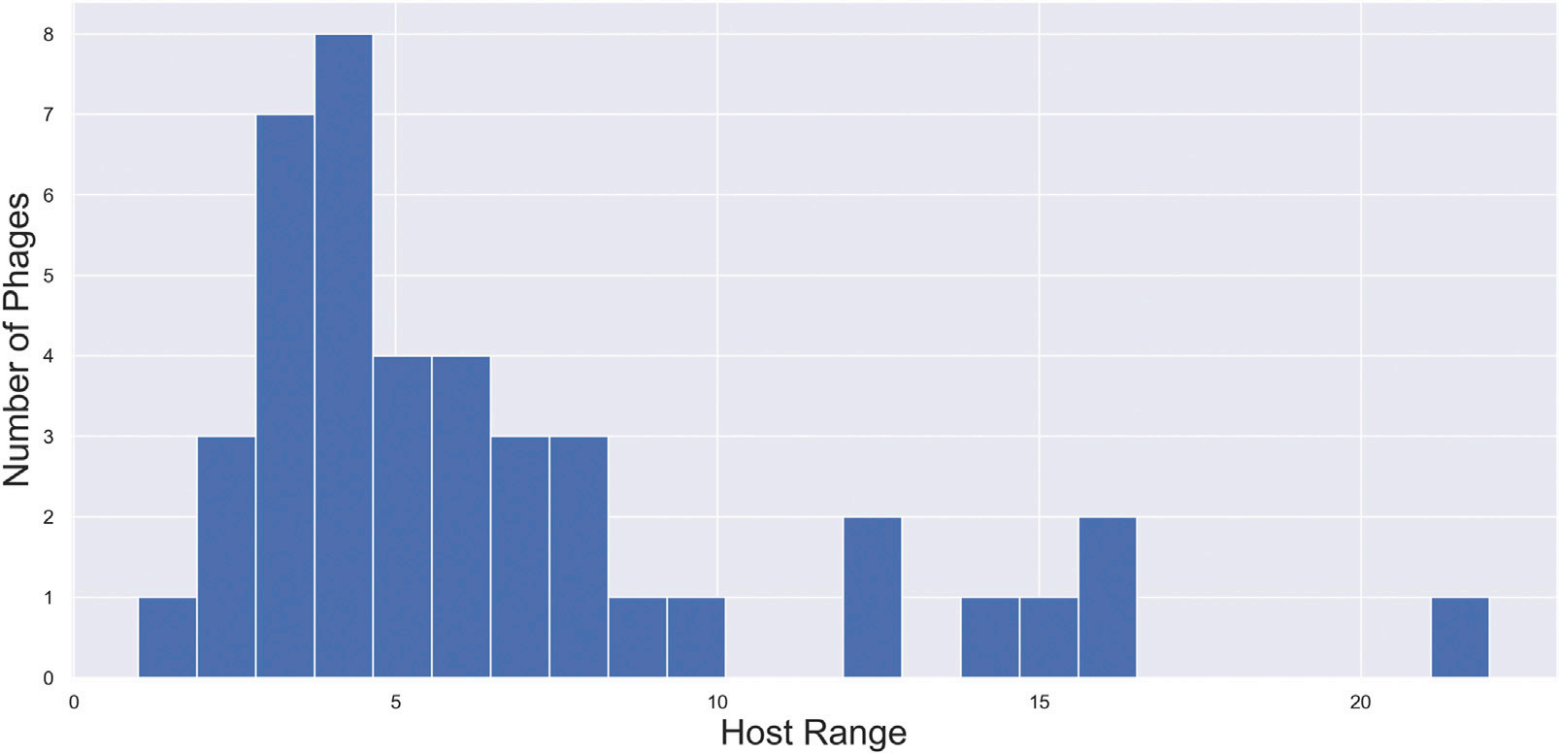
Distribution of host ranges of phages under study. Distribution of the host range of the selected phages across the 46 bacterial hosts available in the dataset. Note that the host range takes on values between 1 and 22, but only 7 of the 42 phages can infect more than 10 bacterial strains.

On this basis, the goal of the *PERPHECT Generator* is to modify, or more precisely to rewrite, the genomes of the phages under study in such a way that, after modification, they can infect as many strains as possible from the *Pseudomonas aeruginosa* species. In other words, we want to modify phages to maximize their *host range*. Figure 3 shows the distribution of the host range of the selected phages across the 46 bacterial hosts available in the dataset. Note that the host range takes on values between 1 and 22, but only 7 of the 42 phages can infect more than 10 bacterial strains.

**FIGURE 4:**
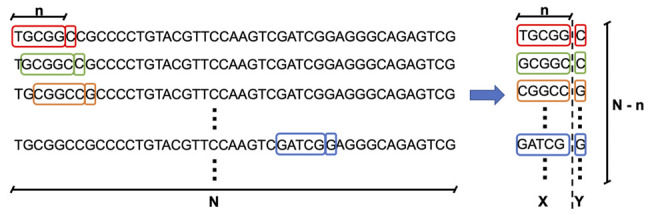
Creation of training data for the generator model. The training data are composed of input–output sub-sequences, where a given input subsequence of length *n* will serve to predict one output symbol, which is the next nucleotide in the sequence. Going through the whole genome sequence of a given phage of length N will, thus, generate (N - n) input–output sub-sequences of length (n+1) symbols. The value n, called seed length, is defined as the number of nucleotide symbols that needs to be passed to the generator model to predict the next nucleotide.

Training the generator model requires providing input–output sub-sequences, where a given input subsequence of length *n* will serve to predict one output symbol, which is the next nucleotide in the sequence. Going through the whole genome sequence of a given phage of length *N* will, thus, generate (*N - n*) input–output sub-sequences of length (*n+1*) symbols, as illustrated in Figure 4. The value *n*, called *seed length*, is defined as the number of nucleotide symbols that needs to be passed to the generator model to predict the next nucleotide. This process is applied to each of the 42 phages in our dataset and repeated for different seed lengths. We use four different values of seed length (200, 500, 700, and 1,000) to build generator models.

From an information point of view, genomic sequences contain four different symbols, representing DNA’s nucleotides (i.e., “A,” “C,” “G,” and “T”). Actual sequences may contain degenerate base symbols which represent more than one potential nucleotide at a given position. As such symbols are very rare in the phage sequences of our dataset, we decided not to keep them as separate symbols but to replace them randomly with one of their possible representations according to the *IUPAC degenerate base symbols* table (Nomenclature, 1970). Finally, the sequences of symbols are *one-hot encoded* to provide an input representation for the neural network. Under such encoding, nucleotides are represented by 4 bits, each bit corresponding to one of the symbols (Brownlee, 2017b).

### 2.2 The Model Architecture

**FIGURE 5:**
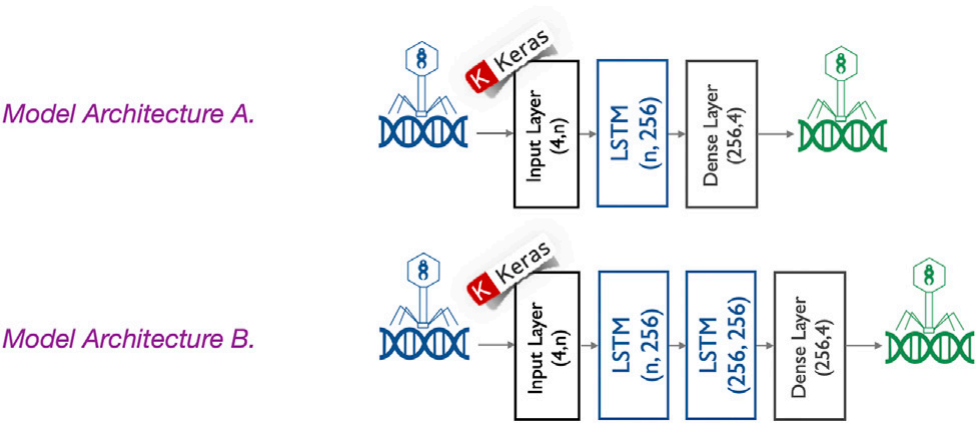
Different generator model architectures. Two different model architectures are proposed. They are composed of an input layer, one or two hidden layers, and an output layer. The input layer takes sequences with n positions (where n = seed length) of four features, each counting for the one-hot encoded input sequences. Each hidden layer is an LSTM with 256 neurons. Architecture A is composed of one hidden layer, whereas the architecture B is composed of two hidden layers. The output layer is a fully connected layer with a softmax activation function to ensure the outputs are interpreted as the probability of membership for each class (i.e., the type of nucleotide).

The proposed generator model is composed of an input layer, one or two hidden layers, and an output layer, as shown in Figure 5. The *input layer* takes sequences with *n* positions (where *n* = *seed length*) of four features, each counting for the one-hot encoded input sequences. Each *hidden layer* is an LSTM with 256 neurons. The *output layer* is a fully connected layer with *softmax* activation function to ensure the outputs are interpreted as the probability of membership for each class (i.e., the type of nucleotide). For this multi-class classification problem, we use commonly used parameters including *cross-entropy* as a loss function (Brownlee, 2020) and an *Adam optimization algorithm* (Brownlee, 2021). To avoid underfitting and overfitting during training, the number of epochs is considered as a *hyperparameter* and tuned using *grid search* while training a few models. The number of epochs is then fixed to the best number of epochs (130 epochs) to avoid underfitting/overfitting, and a *python script* is written to train all different models. Since in our experiments, we trained the proposed deep learning models hundreds of times (e.g., model A is trained 126 times: three times based on the genome sequence of each of the 42 phages under study), we did not use grid search to train all those models. The mean training accuracy and loss of the last three epochs are reported for each trained model. The trained models with a mean accuracy of the last three epochs higher than 70% are also saved. As shown in Figure 5, we propose two different model architectures. They differ in the number of hidden layers but also in the number of phages used to train them. The experiments were developed and run using *Python 3.8.5*, *Keras 2.4.3*, and *TensorFlow-GPU 2.2.0*.

#### 2.2.1 Model Architecture A: A Single LSTM Layer

Model A has a single LSTM with 256 neurons as a hidden layer, and it is trained using genome sub-sequences from only one phage. This phage is selected among the 42 phages under study based on two criteria: i) It should be able to train the generator model with high accuracy and ii) it should have a good enough host range before modification. We trained the generator model A with each bacteriophage separately and repeated the process three times because of the stochastic nature of LSTM models. Then, among those phages whose trained model exhibits a mean accuracy higher than 55%, we selected the phage with the highest host range. Finally, amid the three models of the selected phage, the one with the highest accuracy is chosen as the generator model. As already mentioned, the whole process is repeated four times for the different seed length values: 200, 500, 700, and 1,000.

#### 2.2.2 Model Architecture B: Two Stacked LSTM Layers

Model B has a more complex architecture as its hidden layer is composed of two stacked LSTMs, each with 256 neurons. Thanks to this complexity, it may be used to learn simultaneously from more than one phage genome sequence. Model *A*, with a single LSTM layer, was unable to learn on two or more phage genomes, and considering three or more LSTM layers will imply much more parameters, increasing the risk of overfitting the training data. Next arises the challenge of how to choose a set of two or more phage genomes for training. For this purpose, we apply two criteria: i) each of the selected phage genomes should be able, alone, to train the generator model with high accuracy and ii) their combined host range (i.e., the union of their host sets) should be good enough. To do so, we rank the phages according to their mean accuracy, obtained when learning model *A* and selecting the top phage. Then, we add the next top phage if and only if doing so increases the combined host range. This procedure is repeated for the top 10 candidate phages and guarantees obtaining the highest combined host range with the minimum possible number of phages. Each time a phage is aggregated to the list, we use the selected phages to train model *B*, and because of the stochastic nature of the models, we trained it three times. Finally, from the configuration with the highest mean accuracy, we selected the most accurate model as the final generator to be used to modify genome sequences. This process is repeated for the four different seed length values.

### 2.3 Modifying Phage Genome Sequences

**FIGURE 6:**
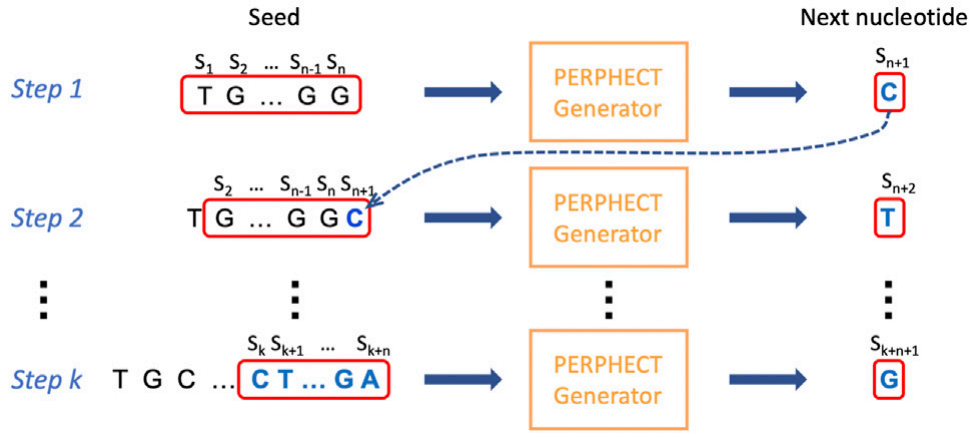
Iterative generation of a genomic sequence. Rewriting phase of the phage genomic sequence by a trained model (A or B). The sequence of the first n symbols (*n* = seed length) is used as input to the model to start the generation process. The input sequence is processed by the model to generate the next character in the sequence. Then, at each iteration, the last n symbols of this growing sequence are passed to the model to generate the next symbol. The process continues until the desired sequence length is attained.

In this phase, the trained model (either model *A* or model *B*) is used to rewrite the genome sequence of all the 42 phages following the iterative process shown in Figure 6. For each phage, we need to provide the sequence of the first *n* symbols (where *n* = *seed length*) as input to the model to start the generation process. The input sequence is processed by the model to generate the next character in the sequence. Then, at each iteration, the last *n* symbols of this growing sequence are passed to the model to generate the next symbol. The process continues until the desired sequence length is attained. In this article, the phage genome sequences are completely rewritten, conserving their original length (comprised between 50,000 and 150,000 nucleotides). Note that as the generator model is trained to very closely reproduce the phage sequences used for training, the generated sequences are expected to be very similar to the original phages.

## 3 Results

The results of the phage genomic modification using the generator models *A (single LSTM)* and *B (two-stacked LSTMs)* are shown in Table 1. For both models and for the four seed lengths used, the following results are presented: the number and ID of the phages used to train the model together with the training accuracy, the number of phages whose host range has been extended, the percentage of host range improvement, the percentage of the phage genome that is modified and, finally, the maximum host range obtained with the corresponding generator. The proposed generators *A* and *B* are trained with an average accuracy of 94.3 and 98.7%, respectively, and are able to improve the host range for, respectively, an average of 18 and 17 phages among the 42 under study. The average host range of phages modified with model *A* is improved by 73%, while with model *B*, it is improved by 103.7%.

**TABLE 1:** Summary of evaluation results for models A and B with four different seed length values. For both models and for the four seed lengths used, the following results are presented: the number and ID of the phages used to train the model together with the training accuracy, the number of phages whose host range has been extended, the percentage of host range improvement, the percentage of the phage genome that is modified, and finally, the maximum host range obtained with the corresponding generator.

Architecture	Seed length	No. of training phages	Training phage IDs	Training accuracy	No. of phages with host range improvement (total = 42)	Average host range improvement (%)	Maximum modified host range obtained
A	200	1	[5,294]	0.89	13	75.5	21
		1	[5,296]	**0.97**	13	57.1	**24**
	500	1	[5,309]	0.95	20	**100**	22
		1	[5,284]	0.92	19	61	21
	700	1	[5,286]	**0.97**	20	63.1	21
	1,000	1	[5,294]	0.96	**22**	81.2	22
B	200	Max: 3	[5,294, 5,296, 5,311]	0.98	10	53.5	22
		Selected: 3					
	500	Max: 5	[5,309, 5,284]	0.99	9	61.3	24
		Selected: 2					
	700	Max: 6	[5,286, 5,296, 5,319, 5,318, 5,323, 5,291]	0.98	24	100	23
		Selected: 6					
	1,000	Max: 5	[5,294, 5,317, 5,291]	**0.995**	**26**	**200**	**29**
		Selected: 3					

The best obtained results are shown in bold.

**FIGURE 7:**
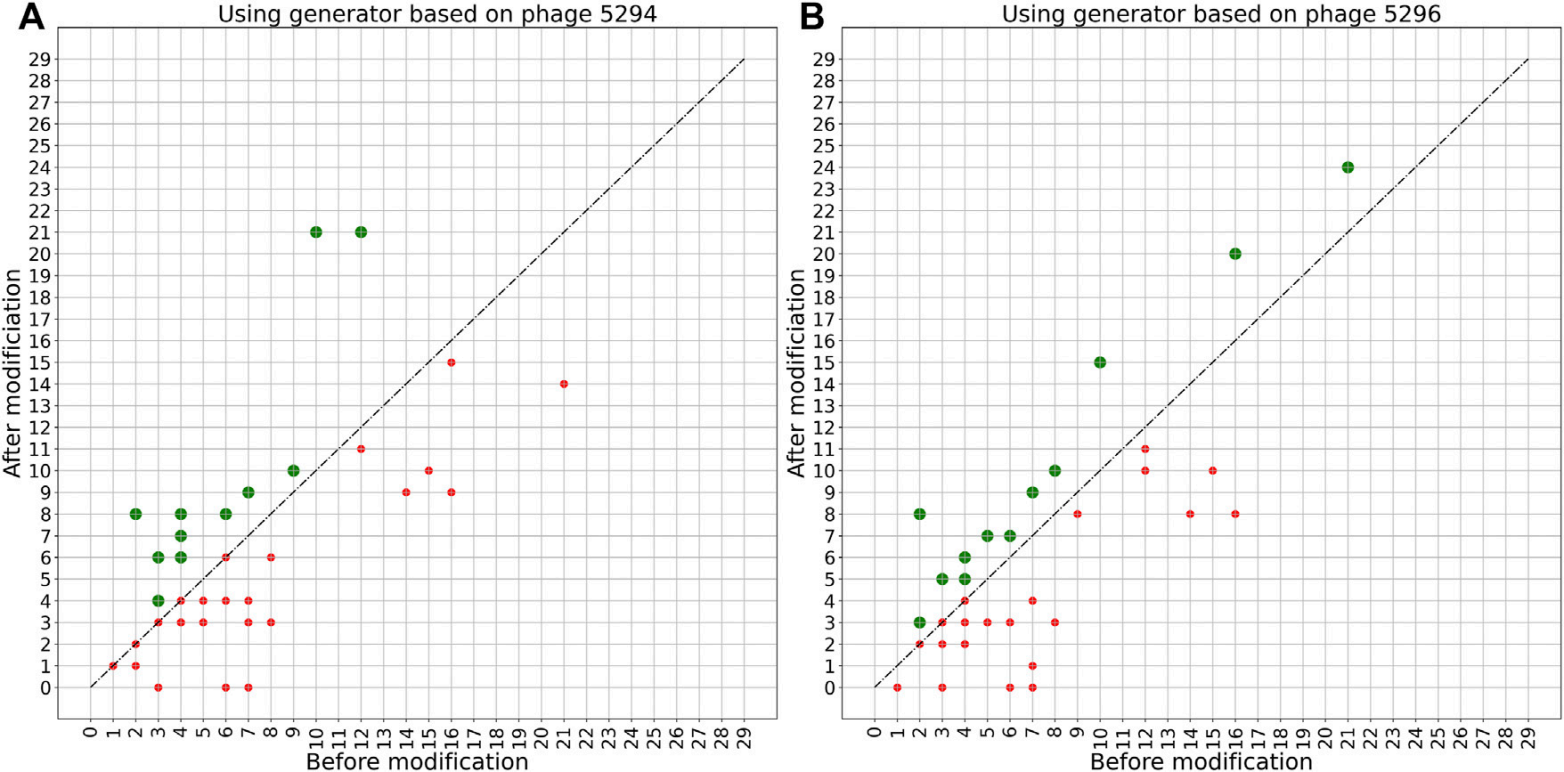
Impact of applying model A with a 200-nucleotide seed on phage sequences. Two models are trained based on the genome sequence of either phage 5294 **(A)** or phage 5296 **(B)** with a seed length of 200. These trained models are then used to modify the genome sequence of all 42 phages under study. The plots show the host range of these phages before and after modification using each trained model. The green spots show phages whose host range is improved after modification. The host ranges of 13 phages are improved after modifying with these two models.

**FIGURE 8:**
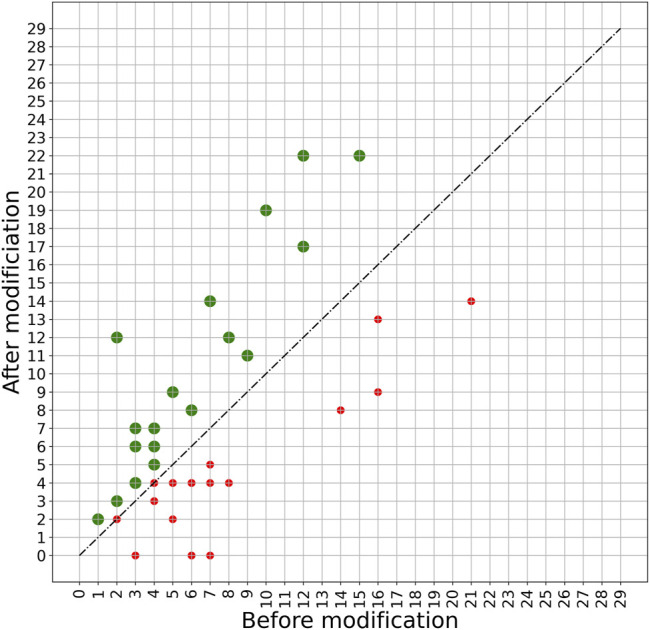
Impact of applying model A with a 1000-nucleotide seed on phage sequences. The model is trained based on the genome sequence of phage 5294 with a seed length of 1000. The trained model is then used to modify the genome sequence of all 42 phages under study. The plot shows the host range of these phages before and after modification. The green spots show phages whose host range is improved after modification. The host ranges of 22 phages are improved after modifying with this model.

**FIGURE 9:**
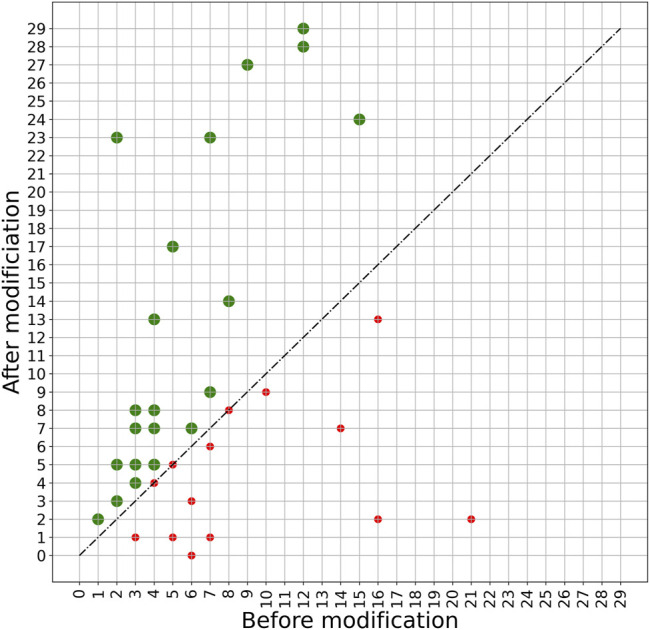
Impact of applying model B with a 1000-nucleotide seed on phage sequences. The model is trained based on the genome sequence of three phages with IDs 5294, 5317, and 5291 with a seed length of 1000. The trained model is then used to modify the genome sequence of all 42 phages under study. The plot shows the host range of these phages before and after modification. The green spots show phages whose host range is improved after modification. The host ranges of 26 phages are improved after modifying with this model.

A more detailed view of the effect of the PERPHECT generator on the host range of all the 42 phages is illustrated in Figures 7–9 for three configurations of interest (Note: The Supplementary Figures S1–S8 present these results for all the configurations explored). In these graphics, green dots represent phages whose host range is improved after modification. Figure 7 shows the configuration exhibiting a maximum host range of 24, the highest for model *A*. It is obtained with *n* = 200. Figure 8 presents the behavior of model *A* with a seed of 1,000 nucleotides. This configuration allows model *A* to improve the host range for 22 of the 42 phages under study, although the maximum host range is only 22. Finally, Figure 9 illustrates the behavior of the best configuration overall: model *B* (*Two stacked LSTMs*) with a seed of 1000 nucleotides which was trained on sequences from three phages. As shown in Table 1, it improves the host range of 26 of the 42 phages under study. This generator is also able to improve the maximum host range from 21 to 29 (a 38% improvement) with six of the phages exceeding 20 predicted interactions.

In general, one can observe from these results that there is no, or little, correlation between the host ranges before and after the modification of the phages. Indeed, the largest host ranges after modification are not necessarily obtained with phages exhibiting the largest host range before modification. From the results, one can associate a higher model complexity (i.e., a stacked LSTM model, trained on several phages, and using longer seeds) with better performance.

## 4 Concluding Remarks

In this article, we proposed an innovative approach composed of two fundamental deep learning components: a phage–bacterium interaction predictor and a phage genome-sequence generator. For the latter component, we developed two different artificial RNN models composed of either one or two stacked LSTM layers with 256 neurons each, while the genome sequence of either one or several phages participated, respectively, in the process of training the models. The proposed generators are used to engineer the genome sequences of 42 selected phages. The phage–bacterium interaction predictor, composed of a multi-branch 1D-CNN, is then used to estimate the host range of the modified bacteriophages across 46 strains of the *Pseudomonas aeruginosa* species under study.

As shown by the evaluation results presented in Table 1 and Figures 7–9, both generator architectures are able to significantly improve the host range of the phage panel under study. For model *A*, based on a single LSTM hidden layer, the seed length used does not seem to have a strong effect on its performance and the most limiting issue appears to be its relatively limited learning capabilities. For model *B*, with two stacked LSTM hidden layers and trained on several phage genomes, the effect of the seed length is more apparent as the host range improvement for seeds of 700 and 1,000 nucleotides is markedly better with respect to the number of improved phages and the average improvement. Nevertheless, the effect on the maximum host range is substantially better only for the longest seed attaining 29. Exploring even the longest seeds could not provide additional advantages as the training accuracy of this model is already 99.5%. These encouraging performances give a green light to further usage of deep learning models, especially LSTM models, in guiding genetic editing of phages to improve their antibacterial power.

Our proposed approach is the first step toward a systematic method to guide the search for genetically engineered phages. Nevertheless, our approach is currently addressing one optimization goal, that is, maximizing phage interaction with target bacteria, while other fundamental criteria, such as minimizing noise, repeats, and non-informative code and maintaining the biological coherence of the organism are yet to be addressed. Some of them, such as noises, repeats, and even unnecessary code, could be tackled by including them in the loss function during the optimization process. Nevertheless, reinforcing the biological coherence of the resulting sequence would require more elaborated strategies. For example, the use of XAI (explainable AI) methods could allow to systematically detect phage-relevant hotspots/motifs in the predictor model. On this base, only essential code segments, selected on the basis of sequence similarity and alignment, will be allowed to integrate existing phage genomes.

## Data Availability

Full data will be released as part of a separate study. However, the source codes for data analysis and ML/DL models can be found here: zenodo, 10.5281/zenodo.6448195. Further inquiries can be directed to the corresponding author.
