# Supplementary material for: Bacteriophage Genetic Edition Using LSTM
Source: Front Bioinform. 2022 Jul 13;2:932319. doi: 10.3389/fbinf.2022.932319 (PMC9639385; doi:10.3389/fbinf.2022.932319)
Supplement: Supplementary file 1 [file DataSheet1.PDF]

Supplementary Material

Detailed view of the effect of the PERPHECT generator on the host range of all the 42 phages. Model A has a single LSTM with 256 neurons as hidden layer, and it is trained using genome sub-sequences from only one phage. Model B has a more complex architecture as its hidden layer is composed of two stacked LSTMs, each with 256 neurons. In these graphics, green dots represent phages whose host range is improved after modification.

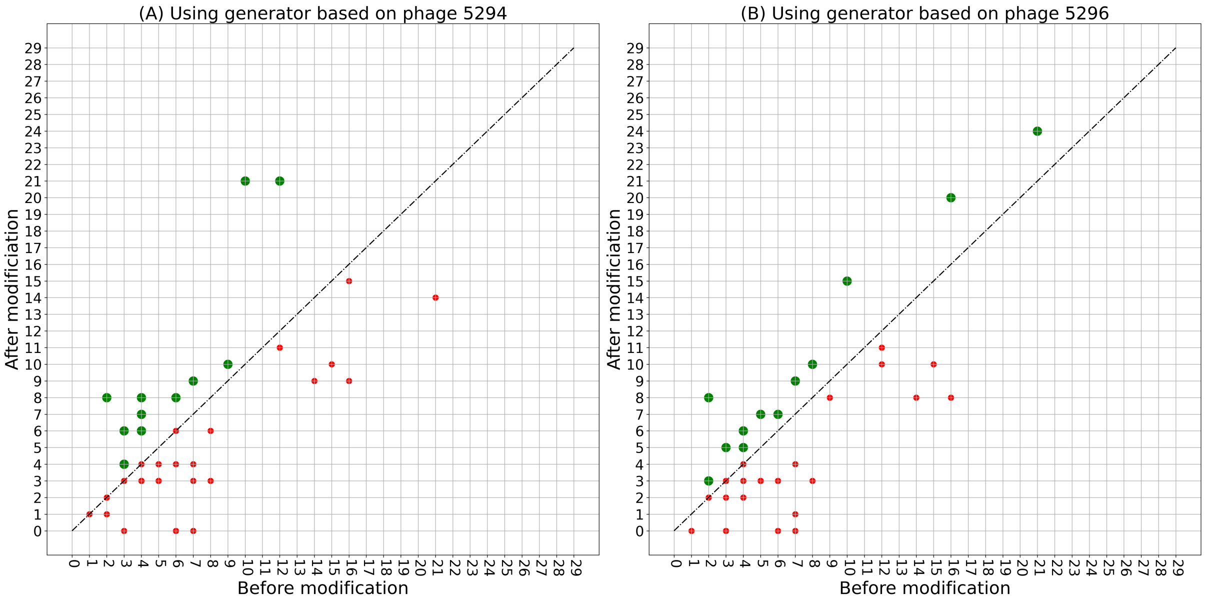

Supplementary Figure 1. Impact of applying model *A* with a 200-nucleotide seed on phage sequences

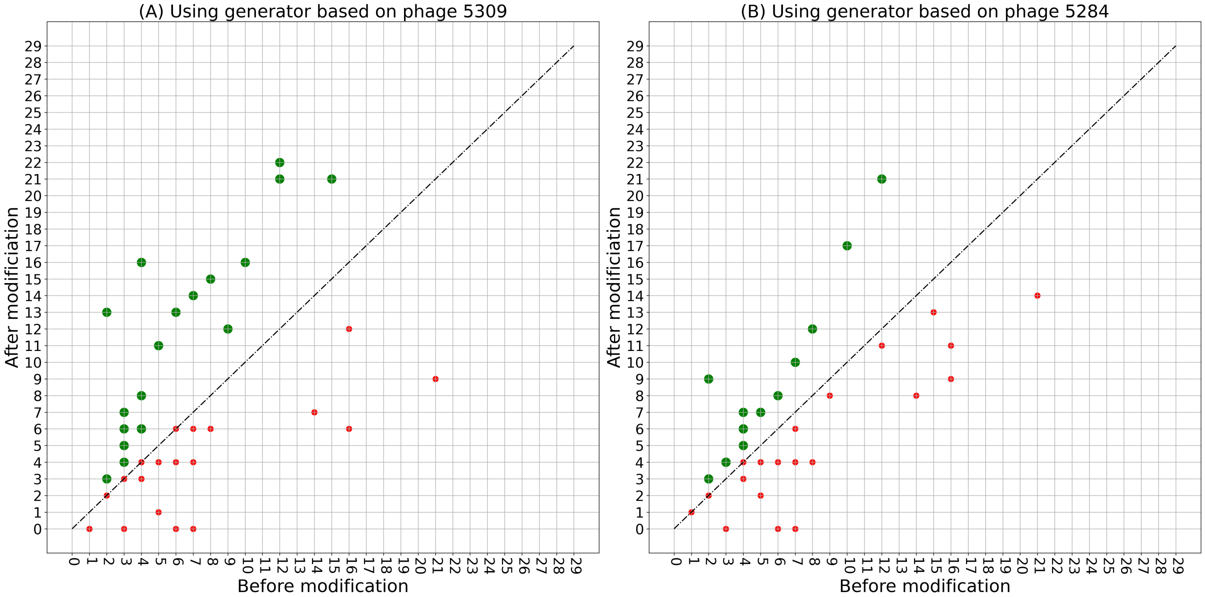

Supplementary Figure 2. Impact of applying model *A* with a 500-nucleotide seed on phage sequences

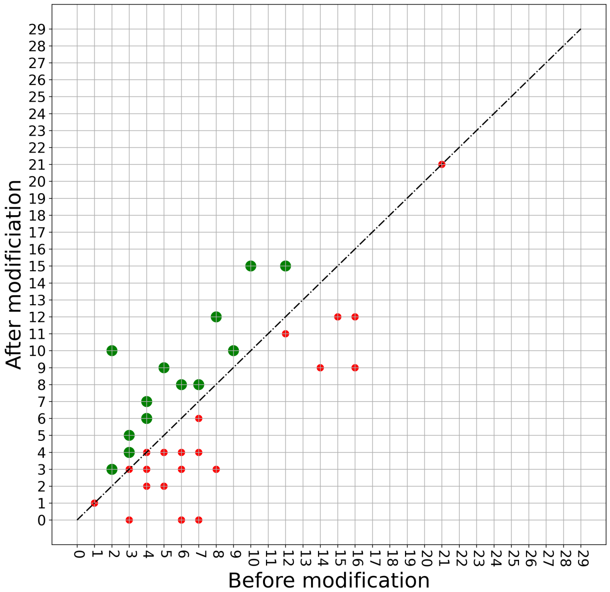

Supplementary Figure 3. Impact of applying model *A* with a 700-nucleotide seed on phage sequences

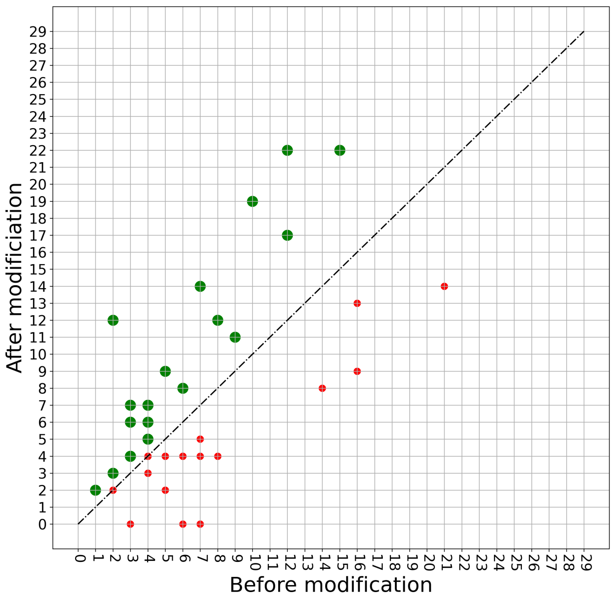

Supplementary Figure 4. Impact of applying model *A* with a 1000-nucleotide seed on phage sequences

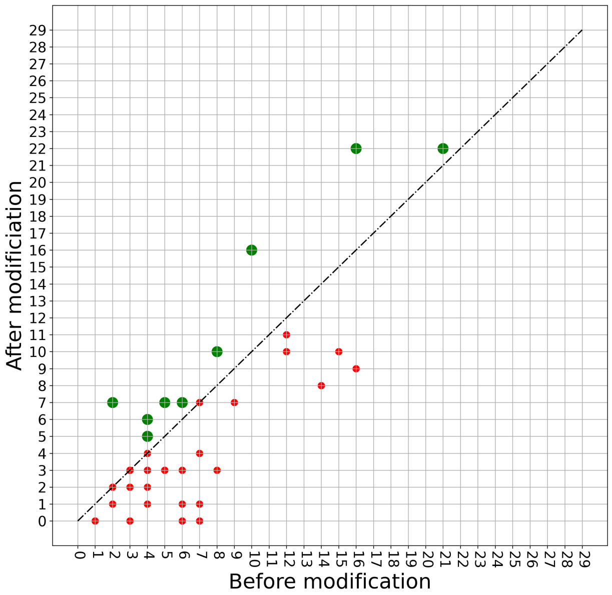

Supplementary Figure 5. Impact of applying model *B* with a 200-nucleotide seed on phage sequences

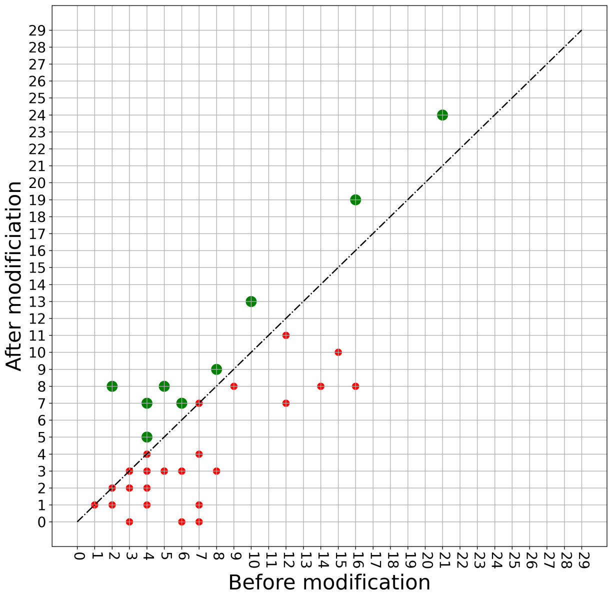

Supplementary Figure 6. Impact of applying model *B* with a 500-nucleotide seed on phage sequences

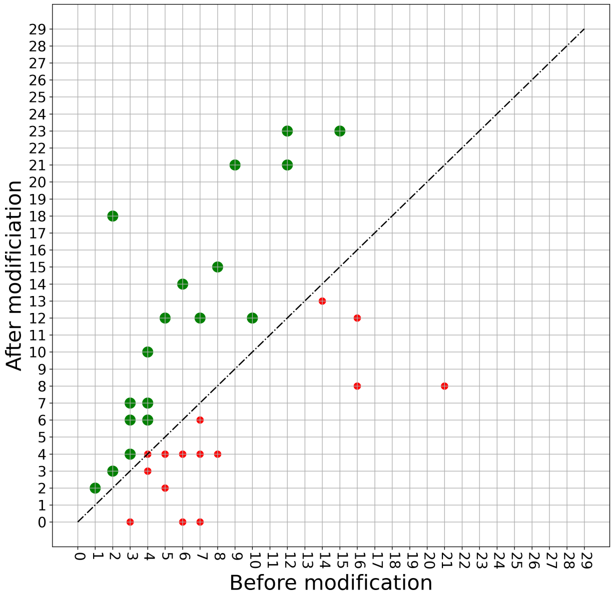

Supplementary Figure 7. Impact of applying model *B* with a 700-nucleotide seed on phage sequences

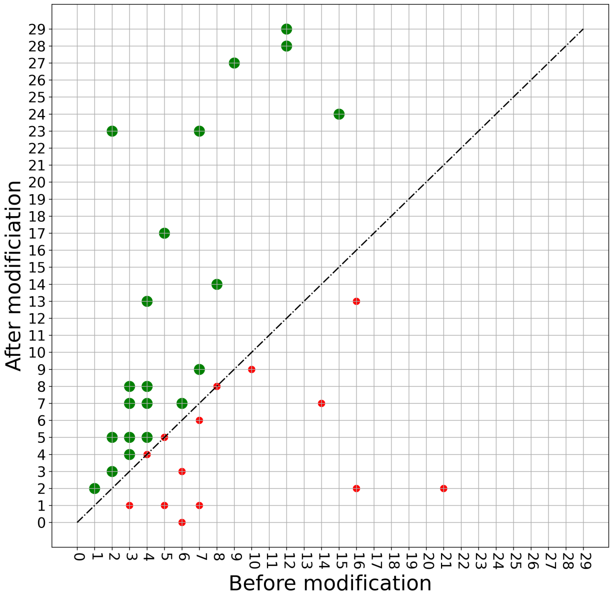

Supplementary Figure 8. Impact of applying model *B* with a 1000-nucleotide seed on phage sequences
